# Supplementary material for: Hot spot prediction in protein-protein interactions by an ensemble system
Source: BMC Syst Biol. 2018 Dec 31;12(Suppl 9):132. doi: 10.1186/s12918-018-0665-8 (PMC6311905; doi:10.1186/s12918-018-0665-8)
Supplement: Supplementary file 2 — All relevant MATLAB code. A simple Matlab implement of our predictor. (ZIP 19,742 kb) [file 12918_2018_665_MOESM2_ESM.zip › Additional file 2/Read ASA/ASA Property/ASEdb/1BRSA.txt]

 NetSurfP - Protein Surface Accessibility and Secondary Structure Predictions

  

|  |  |  |
| --- | --- | --- |
|  |  | NetSurfP - Protein Surface Accessibility and Secondary Structure Predictions  Technical University of Denmark |

  


---

```
# For publication of results, please cite:
# A generic method for assignment of reliability scores applied to solvent accessibility predictions.
# Bent Petersen, Thomas Nordahl Petersen, Pernille Andersen, Morten Nielsen and Claus Lundegaard
# BMC Structural Biology 2009, 9:51 doi:10.1186/1472-6807-9-51
#
# Column 1: Class assignment - B for buried or E for Exposed - Threshold: 25% exposure, but not based on RSA
# Column 2: Amino acid
# Column 3: Sequence name
# Column 4: Amino acid number
# Column 5: Relative Surface Accessibility - RSA
# Column 6: Absolute Surface Accessibility
# Column 7: Z-fit score for RSA prediction
# Column 8: Probability for Alpha-Helix
# Column 9: Probability for Beta-strand
# Column 10: Probability for Coil
E A  1BRSA                  1    0.860  94.794  -0.437   0.016   0.005   0.979
E Q  1BRSA                  2    0.657 117.376   0.030   0.339   0.016   0.645
E V  1BRSA                  3    0.434  66.690   0.099   0.455   0.046   0.498
B I  1BRSA                  4    0.249  46.120  -0.027   0.522   0.016   0.462
E N  1BRSA                  5    0.505  73.888  -0.254   0.522   0.016   0.462
E T  1BRSA                  6    0.404  56.049   0.622   0.430   0.016   0.555
B F  1BRSA                  7    0.132  26.492   0.065   0.858   0.002   0.139
E D  1BRSA                  8    0.550  79.212   0.653   0.923   0.002   0.076
E G  1BRSA                  9    0.430  33.865   0.683   0.923   0.002   0.076
B V  1BRSA                 10    0.030   4.580  -0.041   0.970   0.001   0.030
B A  1BRSA                 11    0.065   7.163  -0.299   0.970   0.001   0.030
E D  1BRSA                 12    0.502  72.381   1.465   0.970   0.001   0.030
B Y  1BRSA                 13    0.137  29.362   1.039   0.970   0.001   0.030
B L  1BRSA                 14    0.023   4.230   0.424   0.970   0.001   0.030
E Q  1BRSA                 15    0.411  73.405   1.051   0.923   0.002   0.076
E T  1BRSA                 16    0.701  97.229   0.794   0.858   0.002   0.139
E Y  1BRSA                 17    0.347  74.261   0.029   0.406   0.004   0.590
E H  1BRSA                 18    0.518  94.224  -0.584   0.053   0.005   0.942
E K  1BRSA                 19    0.458  94.211   0.666   0.016   0.005   0.979
B L  1BRSA                 20    0.088  16.076   0.455   0.016   0.005   0.979
B P  1BRSA                 21    0.204  28.962   0.159   0.016   0.005   0.979
E D  1BRSA                 22    0.577  83.131  -0.860   0.058   0.017   0.925
E N  1BRSA                 23    0.427  62.454  -0.336   0.118   0.150   0.732
B Y  1BRSA                 24    0.207  44.215   0.334   0.148   0.418   0.435
B I  1BRSA                 25    0.078  14.430   0.093   0.074   0.484   0.442
E T  1BRSA                 26    0.263  36.534   0.695   0.066   0.296   0.638
E K  1BRSA                 27    0.427  87.793   1.053   0.717   0.014   0.269
E S  1BRSA                 28    0.619  72.594   0.994   0.858   0.002   0.139
E E  1BRSA                 29    0.394  68.832   0.502   0.923   0.002   0.076
B A  1BRSA                 30    0.082   9.025  -0.842   0.879   0.010   0.111
E Q  1BRSA                 31    0.577 103.034   1.065   0.879   0.010   0.111
E A  1BRSA                 32    0.754  83.113   0.764   0.782   0.003   0.216
E L  1BRSA                 33    0.313  57.292   0.606   0.406   0.004   0.590
E G  1BRSA                 34    0.583  45.898  -1.728   0.109   0.005   0.886
B W  1BRSA                 35    0.124  29.846  -0.765   0.058   0.017   0.925
E V  1BRSA                 36    0.540  82.952   1.162   0.018   0.019   0.964
E A  1BRSA                 37    0.308  33.964  -1.511   0.018   0.019   0.964
E S  1BRSA                 38    0.593  69.558  -1.270   0.018   0.019   0.964
E K  1BRSA                 39    0.582 119.738  -0.223   0.058   0.017   0.925
E G  1BRSA                 40    0.334  26.270  -1.641   0.058   0.017   0.925
E N  1BRSA                 41    0.438  64.196  -0.645   0.058   0.017   0.925
B L  1BRSA                 42    0.158  28.948  -1.361   0.113   0.043   0.844
E A  1BRSA                 43    0.298  32.851  -0.760   0.191   0.086   0.723
E D  1BRSA                 44    0.631  90.956   0.133   0.191   0.086   0.723
B V  1BRSA                 45    0.206  31.647  -0.518   0.113   0.087   0.800
B A  1BRSA                 46    0.148  16.266   0.207   0.053   0.043   0.903
E P  1BRSA                 47    0.528  74.923  -0.357   0.018   0.019   0.964
E G  1BRSA                 48    0.556  43.718  -2.055   0.005   0.015   0.979
E K  1BRSA                 49    0.535 109.988  -0.360   0.018   0.047   0.935
E S  1BRSA                 50    0.426  49.915  -1.234   0.019   0.141   0.840
B I  1BRSA                 51    0.233  43.049  -0.742   0.020   0.205   0.775
E G  1BRSA                 52    0.416  32.763  -1.458   0.019   0.141   0.840
B G  1BRSA                 53    0.194  15.252  -2.175   0.064   0.216   0.721
E D  1BRSA                 54    0.335  48.230  -0.623   0.021   0.451   0.528
E I  1BRSA                 55    0.288  53.280   1.000   0.023   0.655   0.322
B F  1BRSA                 56    0.098  19.729  -0.004   0.022   0.552   0.426
E S  1BRSA                 57    0.344  40.375  -0.027   0.022   0.359   0.619
B N  1BRSA                 58    0.179  26.191  -1.195   0.064   0.216   0.721
E R  1BRSA                 59    0.407  93.249  -0.294   0.064   0.216   0.721
E E  1BRSA                 60    0.438  76.466  -1.005   0.056   0.142   0.802
E G  1BRSA                 61    0.449  35.336  -0.617   0.056   0.142   0.802
E K  1BRSA                 62    0.367  75.389  -0.119   0.056   0.142   0.802
B L  1BRSA                 63    0.158  28.893   0.444   0.052   0.084   0.864
B P  1BRSA                 64    0.147  20.817  -0.846   0.018   0.047   0.935
E G  1BRSA                 65    0.635  49.967   0.478   0.018   0.019   0.964
E K  1BRSA                 66    0.478  98.304   0.346   0.018   0.019   0.964
E S  1BRSA                 67    0.729  85.392  -0.153   0.018   0.019   0.964
E G  1BRSA                 68    0.639  50.289  -0.946   0.005   0.015   0.979
E R  1BRSA                 69    0.368  84.295  -0.529   0.004   0.085   0.910
E T  1BRSA                 70    0.296  41.014   1.395   0.004   0.616   0.381
B W  1BRSA                 71    0.113  27.177   0.663   0.002   0.816   0.182
B R  1BRSA                 72    0.184  42.113   1.476   0.001   0.900   0.099
B E  1BRSA                 73    0.213  37.281  -0.643   0.001   0.900   0.099
B A  1BRSA                 74    0.085   9.389   0.943   0.002   0.816   0.182
E D  1BRSA                 75    0.282  40.564   0.687   0.002   0.816   0.182
B I  1BRSA                 76    0.062  11.470   0.703   0.004   0.616   0.381
E N  1BRSA                 77    0.575  84.253   0.296   0.005   0.336   0.660
B Y  1BRSA                 78    0.186  39.791   0.475   0.018   0.088   0.893
E T  1BRSA                 79    0.574  79.614  -0.654   0.018   0.047   0.935
E S  1BRSA                 80    0.542  63.499  -1.571   0.018   0.019   0.964
E G  1BRSA                 81    0.467  36.784  -1.458   0.018   0.047   0.935
E F  1BRSA                 82    0.527 105.749  -0.991   0.018   0.088   0.893
E R  1BRSA                 83    0.284  65.128  -0.235   0.018   0.088   0.893
E N  1BRSA                 84    0.312  45.691  -0.868   0.018   0.047   0.935
B S  1BRSA                 85    0.141  16.513  -1.209   0.004   0.138   0.858
E D  1BRSA                 86    0.309  44.484   0.423   0.004   0.420   0.576
B R  1BRSA                 87    0.081  18.503   0.681   0.002   0.816   0.182
B I  1BRSA                 88    0.053   9.824   0.135   0.001   0.900   0.099
B L  1BRSA                 89    0.034   6.280  -0.263   0.001   0.900   0.099
B Y  1BRSA                 90    0.101  21.520   0.170   0.001   0.900   0.099
B S  1BRSA                 91    0.085   9.927  -1.509   0.004   0.420   0.576
E S  1BRSA                 92    0.546  63.944  -1.146   0.005   0.015   0.979
E D  1BRSA                 93    0.562  81.013  -1.497   0.018   0.019   0.964
E W  1BRSA                 94    0.326  78.331  -0.678   0.019   0.141   0.840
B L  1BRSA                 95    0.219  40.117  -0.140   0.023   0.655   0.322
B I  1BRSA                 96    0.161  29.822   1.360   0.011   0.918   0.071
B Y  1BRSA                 97    0.095  20.216   0.657   0.001   0.900   0.099
B K  1BRSA                 98    0.171  35.113   0.695   0.001   0.900   0.099
B T  1BRSA                 99    0.077  10.624   0.095   0.004   0.616   0.381
E T  1BRSA                100    0.411  56.936  -0.026   0.191   0.086   0.723
E D  1BRSA                101    0.462  66.502  -0.098   0.430   0.016   0.555
B H  1BRSA                102    0.139  25.230   0.461   0.622   0.015   0.363
B Y  1BRSA                103    0.145  30.922   0.392   0.717   0.014   0.269
E Q  1BRSA                104    0.545  97.301   1.420   0.660   0.049   0.291
B T  1BRSA                105    0.237  32.816   0.549   0.660   0.049   0.291
B F  1BRSA                106    0.071  14.290  -0.438   0.701   0.107   0.192
E T  1BRSA                107    0.469  65.106   1.589   0.649   0.163   0.188
E K  1BRSA                108    0.534 109.885   1.438   0.428   0.171   0.402
B I  1BRSA                109    0.224  41.440  -0.396   0.278   0.093   0.628
E R  1BRSA                110    0.759 173.880   0.516   0.016   0.005   0.979

Explain the output.  Go back.
```

---
